# Energy-Efficient Spiking Segmenter for Frame and Event-Based Images

**DOI:** 10.3390/biomimetics8040356

**Published:** 2023-08-10

**Authors:** Hong Zhang, Xiongfei Fan, Yu Zhang

**Affiliations:** 1 State Key Laboratory of Industrial Control Technology, College of Control Science and Engineering, Zhejiang University, Hangzhou 310027, China; hongzhang99@zju.edu.cn (H.Z.); xffan@zju.edu.cn (X.F.); 2 Key Laboratory of Collaborative Sensing and Autonomous Unmanned Systems of Zhejiang Province, Hangzhou 310027, China

**Keywords:** neuromophic computing, spiking neural network, semantic segmentation, spiking context guided network, frame and event-based images

## Abstract

Semantic segmentation predicts dense pixel-wise semantic labels, which is crucial for autonomous environment perception systems. For applications on mobile devices, current research focuses on energy-efficient segmenters for both frame and event-based cameras. However, there is currently no artificial neural network (ANN) that can perform efficient segmentation on both types of images. This paper introduces spiking neural network (SNN, a bionic model that is energy-efficient when implemented on neuromorphic hardware) and develops a Spiking Context Guided Network (Spiking CGNet) with substantially lower energy consumption and comparable performance for both frame and event-based images. First, this paper proposes a spiking context guided block that can extract local features and context information with spike computations. On this basis, the directly-trained SCGNet-S and SCGNet-L are established for both frame and event-based images. Our method is verified on the frame-based dataset Cityscapes and the event-based dataset DDD17. On the Cityscapes dataset, SCGNet-S achieves comparable results to ANN CGNet with 4.85 × energy efficiency. On the DDD17 dataset, Spiking CGNet outperforms other spiking segmenters by a large margin.

## 1. Introduction

Semantic segmentation is one of the essential tasks in computer vision and has a wide range of applications in autonomous driving, mobile robotics, virtual reality, image editing, intelligent medicine, and other fields [1,2,3]. Semantic segmentation aims to perform dense semantic label prediction for all pixels in an image and provide high-level semantic representations for other tasks. Segmentation methods based on artificial neural networks (ANNs) have become mainstream since the fully convolutional network (FCN) [4] was proposed for solving semantic segmentation problems.

With the widespread interest in autonomous mobile robots, autonomous driving technologies, and other edge computing scenarios, the existing semantic segmentation research has the following trends. The first trend is lightweight and low-power segmentation networks. It is difficult for mobile computing devices to support large-scale, high-energy network operations, and low-power networks are preferred for such scenarios. The second trend is semantic segmentation systems based on event cameras. Event cameras, or dynamic vision sensors (DVS) [5], mainly record the light intensity changes in the environment and have many advantages over traditional frame-based cameras, including high temporal resolution, high dynamic range, and low power consumption. Event cameras have higher robustness in some special cases, such as motion blur and low-light scenes. Currently, there is no method that can complete the high-performance semantic segmentation of both frame and event-based images with low power consumption.

For addressing the above low-power requirement and the capability of adapting DVS data, a segmentation algorithm based on spiking neural networks (SNNs) is a good choice. The spiking neural network is a bionic neuron model inspired by biological neuron models based on spiking signals. Spiking neurons consume energy only when the spike is generated, and this spike activity is usually much sparser than ANNs. Thus, SNN is very energy-efficient when implemented on neuromorphic hardware [6,7]. Meanwhile, SNN has complex temporal dynamics [8], and the bionic activation-based model fits well with the asynchronous nature of sequential streams from event cameras.

In this paper, the semantic segmentation task is considered from the perspective of direct-training SNNs, in order to accomplish dense semantic predictions for images from both conventional cameras and DVS with low power consumption. In the field of SNNs, ANN-SNN conversion is a commonly used method to deploy SNNs. This conversion uses the integrate-and-fire (IF) neurons to replace the rectified linear unit (ReLU) activation function in a pre-trained ANN [9], thereby replacing the whole network with a SNN. Additionally, the corresponding SNN can usually achieve an accuracy comparable to the ANN. However, the SNNs obtained by this approach usually require thousands of time steps, resulting in a high-latency network contrary to the original intention [10,11,12]. Further, the ANN-SNN conversion needs to ensure that the structures and input–output patterns of ANN and SNN are identical. This leads to the inability to complete the training on the DVS dataset and limits the deployment of the method on neuromorphic systems [13]. Therefore, this paper adopts a direct-training approach using backpropagation to optimize the parameters of the SNN directly. The direct-training method expands the SNN through time dimension and adopts backpropagation through time framework (BPTT) [14] for backpropagation. To overcome the non-differentiable characteristic of the step function in spiking neurons, the surrogate gradient function is used [15] to replace the gradient and complete the gradient propagation. The direct-training method can adopt tiny time step configurations [16,17], allowing us to focus on designing the spiking semantic segmentation network structure.

In semantic segmentation or scene understanding tasks, human vision systems tend to recognize the focused pixels with the help of the surrounding context and global scene information [1]. Inspired by the contextual learning of human vision systems, a direct-training spiking context guided network (Spiking CGNet) is designed with substantially lower energy consumption and high performance for both frame and event-based images. This paper adopts the encoder–decoder architecture of ANN CGNet, and modifies the basic module to complete effective contextual learning with only spike computations.

The main contributions of this paper are as follows:

- This paper proposes a spiking context-guided block with spiking neurons and membrane shortcut connections to learn local feature and contextual information under the SNN computing paradigm. Furthermore, to learn global context better, the global context extractor is revised to refine the feature with minimal energy cost.
- This paper designs appropriate input representations and encoding layers for both frame and event-based images, respectively. On this basis, the direct-training Spiking CGNet is established with several modifications from ANN CGNet, including the stem network, multi-scale membrane connection, and the spike decoder.
- This paper validates the performance of Spiking CGNet by comparing it to the ANN and SNN segmenters in the literature on the frame-based Cityscapes dataset and event-based DAVIS driving dataset 2017 (DDD17).

The rest of this paper is structured as follows. In Section 2, the ANN-based semantic segmentation methods and recent studies on spiking neural networks are reviewed. In Section 3, the critical technologies in Spiking CGNet are presented, including input representation, design of spiking context guided block, the whole structure of Spiking CGNet, and the overall training algorithm in this paper. In Section 4, Spiking CGNet is validated on both frame and event-based image datasets. Finally, the conclusion and future works are discussed in Section 5.

## 2. Related Works

### 2.1. Semantic Segmentation

Semantic segmentation aims to perform dense semantic label prediction for all pixels in an image. FCN [4] pioneered using a fully convolutional architecture to identify high-level semantic features of static images. Subsequent works focus on improving FCN and the encoder–decoder architecture. Serveral key techniques have emerged, such as enlarging the receptive field with dilated or atrous convolution [18,19,20], refining the contextual information by multi-scale feature fusion [21,22,23,24], etc. Other researchers have designed lightweight models that take into account both accuracy and resource constraints along with the excellent encoder–decoder, dilated convolution, and multi-scale fusion techniques [1,25,26]. In recent years, the real-time semantic segmentation networks are developing towards high resolution and multi-branching techniques [26,27,28]. Additionally, transfer learning is used to further improve accuracy [29,30]. In this paper, the structure of CGNet is selected as our baseline to explore the issues of spiking semantic segmenter. With the development of the attention mechanism [31], transformer-based segmenters are sprouting up. These works [2,32,33,34] currently demonstrate state-of-the-art performance. Since the spiking transformer [35] is still in the exploration stage, these networks are not considered our baseline.

DVS-based semantic segmentation has also raised lots of attention in recent years. Ev-SegNet [36] accomplished this task using the well-known Xception [37] encoder and a lightweight decoder. They also used a pre-trained model to automatically generate semantic labels for the DDD17 dataset [5]. Subsequent works have improved the results by leveraging additional labeled video datasets [38], event-to-image transfer [39], and unsupervised domain adaptation from still images [40].

### 2.2. Spiking Neural Networks

Unlike ANNs which use analog values for information transfer and calculation, SNNs utilize discrete spikes for conveying information. Combined with the sparsity of activation, SNNs are more energy-efficient than ANNs. Spiking neurons, including IF, LIF [8], PLIF [41], etc., play an essential role in converting analog values into spike ones. There are two ways to obtain deep SNNs, namely ANN-SNN conversion and direct-training. The ANN-SNN conversion methods [9,10,11,12,42,43] use spiking neurons to replace the ReLU activation function in a pre-trained high-performance ANN to obtain a SNN with comparable performance. However, the SNNs obtained by this method typically have high latency and cannot handle DVS data [13]. In the field of direct-training, the backpropagation through time framework is adopted to unfold and train SNNs from scratch [14]. For the non-differentiability of the trigger function in spiking neurons, the surrogate gradient is adopted for error backpropagation [15,44,45]. Further, the issues of residual connection [16,17], batch normalization [46,47], and self-attention [48,49] based on SNNs are explored.

Limited computational resources constrain many application scenarios of downstream vision tasks, and the low-power property of SNNs is well-suited. Currently, SNNs have been applied to several tasks, such as object detection [13,29,50,51,52], optical flow estimation [53,54,55], and object tracking [56,57]. Reference [58] is the first and currently the only SNN work on semantic segmentation. They proposed Spiking FCN and Spiking DeepLab networks based on plain encoder–decoder by referring to the classical structure of ANN segmenters. However, both networks perform poorly on frame and event-based datasets, far from the application. This paper explores the critical issues in semantic segmentation and proposes Spiking CGNet, which is much more energy-efficient and performs comparably to the original CGNet [1].

## 3. Materials and Methods

In this section, after briefly explaining the spiking neuron dynamics as a preliminary, this paper first presents our representations for frame and event-based inputs. Then, the structure of the spiking context-guided block is illustrated, which is the basic module for Spiking CGNet. Next, the structure of ANN CGNet is redesigned to meet the SNN paradigm. Finally, the overall training algorithm in this paper is described.

### 3.1. Spiking Neuron Model

The spiking neuron is the activation function in SNNs and plays a vital role in the conversion and transmission of spiking signals. The discrete-time dynamics of the well-known leaky integrate-and-fire (LIF) [8] neuron can be formulated as follows:

(1) $$H\left[t\right]=V\left[t-1\right]+\frac{1}{\tau_{m}}\left(I\left[t\right]-\left(V\left[t-1\right]-V_{reset}\right)\right)$$

where V[t−1] represents the membrane potential at time t−1, and H[t] is the hidden membrane potential before trigger time *t*. I[t] is the synaptic current. $V_{reset}$ represents the resting potential. Once H[t] exceeds the firing threshold $V_{th}$, the neuron will file a spike expressed as follows:

(2) $$S\left[t\right]=\Theta \left(H\left[t\right]-V_{th}\right)$$

where S[t] denotes the output, and Θ is the Heaviside step function. Then, membrane potential at time *t* will be updated as:

(3) $$V\left[t\right]=H\left[t\right]\left(1-S\left[t\right]\right)+V_{reset}S\left[t\right]$$

In addition to LIF, this paper also uses the IF and parametric leaky integrate-and-fire (PLIF) [41] neurons in this work. Their integration dynamics differ from LIF expressed in Equation (1), while the fire and reset processes remain unchanged. The IF neuron abandons the leakage of membrane voltage, and its integrate dynamics is shown in Equation (4). The PLIF neuron replaces the time constant of LIF with a learnable parameter *a*, thus expanding the network’s learning ability. The discrete-time dynamics of PLIF are shown in Equation (5).

(4) H[t]=V[t−1]+I[t]

(5) $$H\left[t\right]=V\left[t-1\right]+\frac{1}{1+\exp\left(-a\right)}\left(I\left[t\right]-\left(V\left[t-1\right]-V_{reset}\right)\right)$$

In the field, it is generally believed that multiplication operations that contain spike values as inputs are spike computations, such as spike-float and spike-spike multiplications. The multiplications of two non-spike inputs as spike computations are not considered, such as integer-float and float-float multiplications. The general criterion for SNN design is to ensure that the input of the main convolution module is a spike value, which can ensure that the convolution calculation is a spike computation.

### 3.2. Input Representation

Our method can handle two types of data: static and DVS images. For the sake of input consistency, both types of inputs should be converted to representations of dimension T×c×h×w, where *T* is the time step of the SNN, and *c*, *h*, and *w* are the channel numbers, height, and width of static or DVS images.

For a static image whose dimensions are c×h×w, a standard 3×3 convolution is used as the encoding layer. Then this paper copies the encoded image along the time dimension, which means that the encoded images serve as the time-invariant inputs for the subsequent SNN at all time steps.

Raw DVS data are often recorded as an event stream. A single event can be described by a four-value tuple $\left(t,p,x,y\right)$, where *t* is the time at which the event occurred, usually a continuous time value. *p* denotes the polarity of the event, indicating whether the light intensity increases or decreases. In addition, (x,y) indicates the two-dimensional pixel coordinates of the event in the camera. Therefore, an event stream with *N* input events can be represented as $S={\left\{\left(t_{i},p_{i},x_{i},y_{i}\right)\right\}}_{i\in [1,N]}$. For convenience of calculation, continuous time needs to be discretized, and the common way is to divide the time interval into *B* discrete bins. In order to obtain the voxel grid $V\in R^{T,c,h,w}$, the polarity of events is used as the channel dimension, and then discretize *S* to *V* with Equation (6).

(6) $$V\left(t,p,x,y\right)=\sum_{i}k\left(x-x_{i}\right)k\left(y-y_{i}\right)k\left(t-t_{i}^{*}\right)$$

where $t_{i}^{*}=\left(B-1\right)\left(t_{i}-t_{1}\right)/\left(t_{N}-t_{1}\right)$ and k(x) is the bilinear sampling kernel defined as k(x)=max(0,1−|x|). For the DDD17 dataset, this paper accumulates positive and negative events separately, resulting $V\in R^{B,2,h,w}$. To reduce the simulation time step *T* of the network and retain a high time resolution of the data, this paper moves the information of adjacent time bins to the channel dimension. That is, reshape $V\in R^{B,2,h,w}$ to $V\in R^{T,\frac{2B}{T},h,w}$.

### 3.3. Spiking Context Guided Block

**Figure 1:**
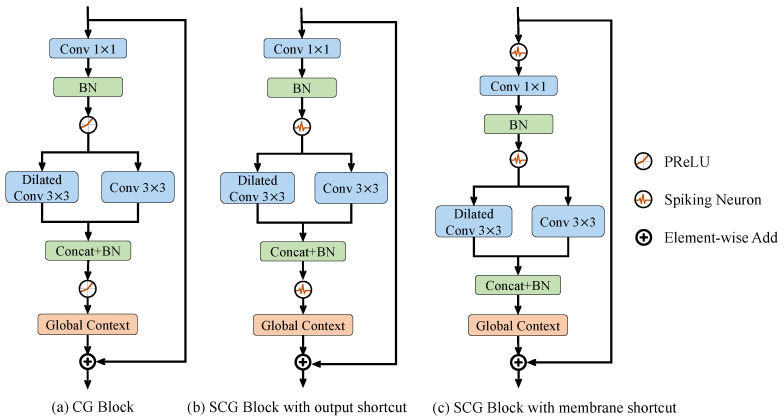
Illustrations of (**a**) CG block, (**b**) SCG block with output shorcut, and (**c**) SCG block with membrane shortcut. CG block is inspired by the human visual system and consists of three convolutional layers and a global context extractor. To convert all operations into spike computation, the PReLU activation functions are first replaced in CG block with spiking neurons. Furthermore, SCG blocks are designed with two kinds of residual connections, the output and membrane shortcuts, as shown in (**b**,**c**). It is proven that only the membrane shortcut can ensure the spiking characteristic of all convolutional operations. Conv 1×1 and BN means the convolutional layer with a 1×1 kernel and the batch normalization layer. Dilated means that the convolution kernel is arranged in a separated way to increase the receptive field. Concat means the concatenation of multiple inputs along the channel dimension.

The spiking context-guided (SCG) block is the basic module of Spiking CGNet. It can efficiently extract local features and contextual information using the SNN computing paradigm. The overall design concept is shown in Figure 1, which plots the ANN context-guided block (Figure 1a) and the two proposed spiking context-guided blocks (Figure 1b and Figure 1c). In semantic segmentation or scene understanding tasks, human vision systems tend to recognize the focused pixels with the help of the surrounding context and global scene information [1]. From a functionality perspective, critical operators in Figure 1 include Conv 1 × 1, Dilated conv 3 × 3, Conv 3 × 3, and Global Context. Conv 1 × 1 is responsible for the projection of features and is also the network’s primary source of complexity growth. Dilated conv 3 × 3 is a surrounding context extractor. Conv 3 × 3 stands for a local feature extractor. Global Context is the global context extractor of the whole image.

Figure 1a shows the human visual-inspired context-guided (CG) block in ANN CGNet. After a 1 × 1 convolutional transformation, it uses standard 3 × 3 convolution and dilated 3 × 3 convolution (which has a larger receptive field) to extract local features and the corresponding surrounding context of the image, respectively. Then, this module combines concatenation and batch normalization to fuse local features with the surrounding context to form joint features. Finally, the global context extractor based on channel-wise attention is adopted to extract the global context of the image and refine the joint features.

#### 3.3.1. SCG Block with Output and Membrane Shortcut

Convolution determines the weight connection between neurons in the previous and current layers, making it suitable for both artificial and spiking neurons. For simple structures such as VGGNet [59], replacing artificial neurons with spiking neurons can convert the network into a SNN. It is worth mentioning that the CG block uses a shortcut structure from input to output to solve the problem of gradient disappearance. By directly replacing the parametric rectified linear unit (PReLU) in Figure 1a with a spiking neuron, the SCG block can be obtained with the output shortcut shown in Figure 1b.

In Figure 1b, the module’s output is a non-spike value because of the scale of the global context extractor and output shortcut. At the same time, as the basic module is sequentially connected, the inputs of all modules (except for the first one) are non-spike. Therefore, the first convolution in the module does not use spike computation, which makes the structure unacceptable.

To address this issue, this paper uses the idea of membrane shortcut to improve the structure of the SCG block, as shown in Figure 1c. It has two key points. First, the shortcut connection is set at the input of the spiking neuron, corresponding to the input membrane of the neuron. Second, a spiking neuron is set before each convolutional layer to ensure the input a spiking signal. In Figure 1c, the inputs of all convolutional layers are the output spikes from spiking neurons, ensuring that all multiplications are spike computations. At the same time, the output of each block serves as the input of the first neuron in the subsequent block, avoiding float-float multiplication, which meets the design criteria of SNN. Therefore, the SCG block with membrane shortcut is suitable as the basic unit used in Spiking CGNet. In the remaining part of this article, the SCG block refers to this structure.

#### 3.3.2. Global Context Extractor

**Figure 2:**
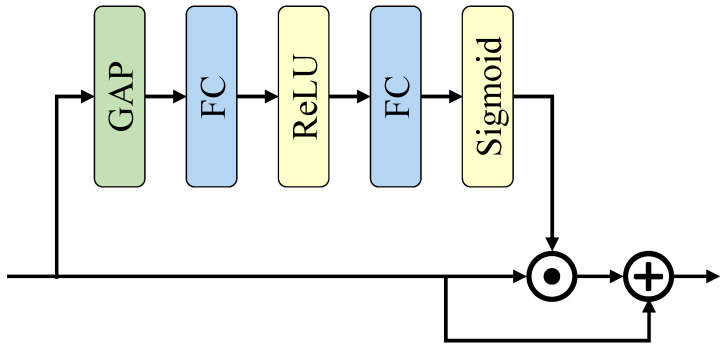
Illustration of global context extractor in SCG block. This paper adopts channel-wise attention to extract the global context of the image and refine the joint features. The GAP is changed to a 3-dimensional global average pooling to process temporal information.

In Figure 1, the SCG block uses a global context extractor (GCE) to refine the joint feature. Its detailed structure is shown in Figure 2. First, a global average pooling is used to squeeze the joint feature along the channel dimension. Then, two fully connected layers are used to extract the global context, which is finally used to refine the joint feature. The reduction ratio *r* is used to reduce the computational cost of the fully connected layers. Consider FC1 and FC2 as the first and second fully connected layers in Figure 2, and assume that the input channels of FC1 and the output channels of FC2 are both *c*. This paper reduces the output channels of FC1 and input channels of FC2 to c/r using the reduction ratio *r*. Therefore, the total computation of the layers is $c\times c/r+c/r\times c=2\times c^{2}/r$, which is *r* times lower than that without reduction, which is $2\times c^{2}$.

Our design has two main differences from the global context extractor in CGNet. First, the module’s input *X* contains an additional time dimension, which means $X\in R^{T\times c\times h\times w}$. *T*, *c*, *h*, and *w* denotes the time step, channel number, height, and width, respectively. Therefore, our global average pooling (GAP) is 3-dimensional for time, width, and height, and the final weight dimension goes to $X\in R^{1\times c\times 1\times 1}$. Second, the convergence of SNN is more complicated than ANN, so this paper adds residual connections in the GCE so that the unrefined features can directly affect the final loss.

Unlike the convolution layers, the non-spike computation is retained in GCE because it only brings minimal computational burden. The total energy consumption of the GCE module is only 2.03% of the entire network, which will be analyzed in detail in Section 4.

### 3.4. Spiking Context Guided Network

**Figure 3:**
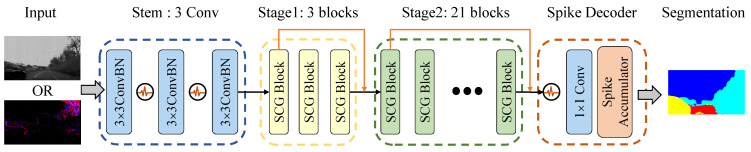
Illustration of the proposed spiking context guided network. It consists of a stem, two stages with SCG blocks, and a spike decoder. The stem takes static images or DVS data as input and output the encoded spiking features. The middle two stages have 3 and 21 SCG blocks, respectively, and the first block of each stage downsamples the feature map by a factor of 2. The spike decoder decodes the spiking features into the final segmentation predictions.

The high-level structure of the Spiking CGNet is shown in Figure 3, which includes a stem, two stages with SCG blocks, and a spike decoder. The stem takes static images or DVS data as input and contains three sequentially placed convolutional layers. The middle two stages have 3 and 21 SCG blocks, respectively, and the first block of each stage downsamples the feature map by a factor of 2. After the two stages, the spike decoder decodes the spiking features into the final segmentation prediction. The overall structure of Spiking CGNet is similar to ANN CGNet. However, to adapt to spike computation and improve segmentation performance, several improvements have been achieved.

Firstly, all PReLU activations of the stem are replaced by spiking neurons. Therefore, the stem not only downsamples the input data but also encodes non-spike inputs into spike features. The first convolutional layer is regarded as the encoding layer. Moreover, the encoding layer varies according to the two types of input data. For static image input, it is a standard 2-dimensional 3×3 convolutional layer. For the voxel representation of event streams input, it changes to a spiking convolutional layer which performs convolution with the same kernel at all time steps.

Secondly, this paper concatenates the output of the first SCG block in the middle stages into the corresponding stage output, shown as the skip connections in orange arrows in Figure 3. This operation can fully utilize the multi-scale features and improve the segmentation accuracy. Moreover, all skip connections use the membrane shortcuts mentioned in Section 3.3. The concatenated inputs are the membrane potentials of spiking neurons, which ensure that the corresponding calculations are all spike computations.

Finally, the spike decoder is redesigned to decode the spiking feature maps to predictions. This paper uses the combination of a spiking neuron and a 1 × 1 convolutional layer to transform the feature maps so that the channel numbers correspond to semantic categories. Then, the spike accumulator calculates the firing rate of the spiking feature maps as the semantic predictions.

By changing the number of channels in Figure 3, two configurations of Spiking CGNet are proposed: SCGNet-S and SCGNet-L, which mean the small and large configurations in model complexity. The channel numbers in the stem, stage1, and stage2 of SCGNet-S are 32, 64, and 128, respectively. Its parameter quantity and accuracy are close to ANN CGNet. All channel numbers of SCGNet-L are twice that in SCGNet-S, making it a model with higher accuracy and more parameters.

### 3.5. Overall Training Algorithm

SCGNet is trained using the direct-training method, and the backward gradient is calculated through the backpropagation through time framework. In error backpropagation, the final output *Q* is determined by the spike decoder, which is:

(7) $$Q=\frac{1}{T}\sum_{t=1}^{T}o^{t}$$

where $o^{t}\in R^{n\times h\times w}$ denote the feature map at time step *t* output by the last 1 × 1 convolutional layer, and *T* is the total time steps. *n* is the number of semantic classes in the dataset, and *h* and *w* are the height and width of the output. Then, this paper makes the output $Q=(q_{1},q_{2},\cdots ,q_{n})$ pass through a softmax layer to obtain the final 2-dimensional probability map. For every pixel location (u,v), the predicted semantic label vector *p* is calculated as follows:

(8) $$p_{i}^{\left(u,v\right)}=\frac{e^{q_{i}^{\left(u,v\right)}}}{\sum_{j=1}^{n}e^{q_{j}^{\left(u,v\right)}}}$$

During training, the loss function is determined as the cross-entropy. Given the predicted label vector $P=(p_{1},p_{2},\cdots ,p_{n})$ and the ground truth $Y=(y_{1},y_{2},\cdots ,y_{n})$, loss function *L* is defined by:

(9) $$L=-\frac{1}{N}\sum_{u,v}\sum_{i}^{n}w_{i}y_{i}^{\left(u,v\right)}\log\left(p_{i}^{\left(u,v\right)}\right).$$

where *N* is a normalization factor, and log stands for logarithmic function with a base of 10. To avoid class imbalance in the training dataset, this paper uses a class weighting scheme [60] defined as $w_{i}=\frac{1}{ln(1.02+ratio_{i})}$, where $w_{i}$ denotes the weight of *l*-th semantic category. This paper restricts the weight to interval [1,50] according to $ratio_{i}$, which is the ratio of the total number of pixels in category *i* to the total number of all pixels.

For the last layer, the gradient of weight parameters can be calculated by the final loss. By applying the chain rule, the gradients of loss *L* with respect to the weight parameter $W_{l}$ at the *l*-th hidden layer can be calculated as follows:

(10) $$\frac{\partial L}{\partial W_{l}}=\sum_{t}\left(\frac{\partial L}{\partial O_{l}^{t}}\frac{\partial O_{l}^{t}}{\partial U_{l}^{t}}+\frac{\partial L}{\partial U_{l}^{t+1}}\frac{\partial U_{l}^{t+1}}{\partial U_{l}^{t}}\right)\frac{\partial U_{l}^{t}}{\partial W_{l}}$$

where $O_{l}^{t}$ and $U_{l}^{t}$ represents the output spike and membrane potential at time step *t*, respectively. Because of the non-differentiable spiking activities, $\frac{\partial O_{l}^{t}}{\partial U_{l}^{t}}$ does not exist in practice. Thus, during training, the arc tangent (ArcTan) function ($\sigma^{\prime }\left(x\right)=\frac{1}{1+{\left(\pi x\right)}^{2}}$) is used as the surrogate function to calculate the gradients of all spiking neurons.

## 4. Results and Discussion

In this section, our Spiking CGNet is verified on the static image dataset Cityscapes and the DVS dataset DDD17. Firstly, the experimental settings are explained, including introducing the two datasets and implementation details. Then, this paper compares Spiking CGNet with some classic real-time semantic segmentation methods on Cityscapes and with similar SNN semantic segmentation networks on the DDD17 dataset to demonstrate the effectiveness of Spiking CGNet. Finally, the network’s energy consumption is theoretically calculated to prove the energy efficiency of Spiking CGNet.

### 4.1. Experimentall Settings

#### 4.1.1. Cityscapes Dataset

Cityscapes [61] is a driving dataset for semantic segmentation consisting of 5000 high-resolution images with 19 categories in street scenes for 50 cities. The dataset contains 2975 images in the training set, 500 images in the validation set, and 1525 images in the test set. The image resolutions are re-scaled to 512 × 1024 for high-performance segmentation. During the training process, standard augmentation strategies are used, including random crop, random flip, photo metric distortion, and normalization. No enhancement is performed during the testing process.

#### 4.1.2. DDD17 Dataset

The DAVIS Driving dataset [5] is an event-stream dataset targeting automotive scenarios. It contains 12 h of driving data recorded with a DAVIS sensor, which provides per-pixel aligned and temporally synchronized events and gray-scale frames. This paper uses the segmentation labels generated by Ev-SegNet [36]. Since the DAVIS only features a low resolution, several classes are fused. Only labels for six merged classes are provided: flat (road and pavement), background (construction and sky), object, vegetation, human, and vehicle. During training and testing, each sample contains 12,000 events, which are converted into voxel grids with a spatial resolution of 260 × 346. After generating the event image represented as voxel grids, this paper performs a random crop and flip with a ratio of 0.5 for a simple augmentation during training.

#### 4.1.3. Implementation Details

In our experiments, all spiking neurons’ implementation and GPU acceleration are based on the PyTorch and SpikingJelly [62] frameworks. This paper adopts the IF and PLIF neurons for the Cityscapes and DDD17 datasets, respectively. The Adam optimizer is used for all experiments with a weight decay of 1 × $10^{-5}$. The “poly” learning rule is adopted with a learning rate of 0.001, a power of 0.9, a batch size of 8, and 160,000 iterations. To reduce GPU memory cost and accelerate training, this paper adopts mixed precision training in PyTorch. During comparison, this paper reports the mean intersection over union (mIoU) metric, which is the averaged value over all classes.

### 4.2. Comparisons on Cityscapes

To verify the effectiveness of spiking CGNet, this paper compares it with several typical real-time semantic segmentation networks from the aspects of parameters, floating point operations (FLOPS), and mIoU. Among them, FLOPS directly reflects the model’s computational complexity. Since SNN and ANN adopt different types of operations, this paper uses multiply-and-accumulate (MAC) and accumulate (AC) to distinguish them here. For example, the input data are all floating-point numbers in the ANN convolutional layer, so the computations are MAC operations. Meanwhile, the input data of SNN convolutional layer are all spiking signals, and the computations belong to AC operation. Moreover, these operations occur only when the spiking neuron fires a spike to save energy. Thus, the corresponding FLOPS of MAC and AC operations can be obtained by Formulas (11) and (12).

(11) $$FLOPS\_\mathrm{MAC}=h\times w\times C_{in}\times C_{out\;}\times k^{2}$$

(12) $$FLOPS\_\mathrm{AC}=h\times w\times C_{in\;}\times C_{out}\times k^{2}\times F_{r}\times T$$

where *h* and *w* are the spatial resolution of the input image, $C_{in}$ and $C_{out}$ denote the input and output channel size, and *k* is the weight kernel size. $F_{r}$ and *T* denote the firing rate and time steps in the SNN. The experimental results are shown in Table 1. For a fair comparison, FLOPS are calculated with a 3×640×640 input.

In terms of efficiency, the SNN model SCGNet-S has the same number of parameters as ANN CGNet [1]. This also proves that the design process from ANN CGNet to SCGNet introduces no other parameters. SCGNet-S has only 0.5 G parameters, which is lower than almost all semantic segmentation networks except for ESPNet [65]. The FLOPS_MAC of SCGNet-S and SCGNet-L models are only 0.1 G and 0.2 G, respectively. Almost all convolution calculations in the network use AC operations, and only a few structures, such as the encoding layer and global context extractors, use MAC operations. All computations used in an ANN are MAC, so the FLOPS_MAC of all ANN-based segmenters is much higher than that of Spiking CGNet. The FLOPS_MAC of the early method SegNet [63] even reaches 286 G. The reduction in FLOPS_MAC leads to the saving in energy consumption, which will be further analyzed in the ablation study.

Spiking CGNet is currently the only SNN segmenter that has been experimentally verified on the Cityscapes dataset. From the mIoU perspective, the result is satisfactory. SCGNet-S achieved a mIoU of 62.5%, which is higher than SegNet [63], ENet [60], and ESPNet [65]. Further, the mIoU of SCGNet-L reached 66.5%, which is 1.7% higher than that of ANN CGNet. As a SNN that values energy efficiency, SCGNet’s network accuracy is indeed challenging to surpass the methods in the recent two years, including BiSeNetV2 [26], CABiNet [30], HyperSeg [66], DDRNet [27] and PIDNet [28]. The high accuracy of these methods mainly lies in two aspects. On the one hand, the backbones of the high-precision networks are usually pretrained on the large-scale dataset [67] to obtain better feature representation [30,66]. On the other, these networks are gradually developing towards high resolution and multi-branching. Their image resolution is much higher than previous methods, such as 2048×1024 [26,27,28]. However, improving accuracy by referring to the above two aspects for SNNs is challenging due to the convergence difficulty, substantial computational cost, high memory usage, etc., [16,17].

**Figure 4:**
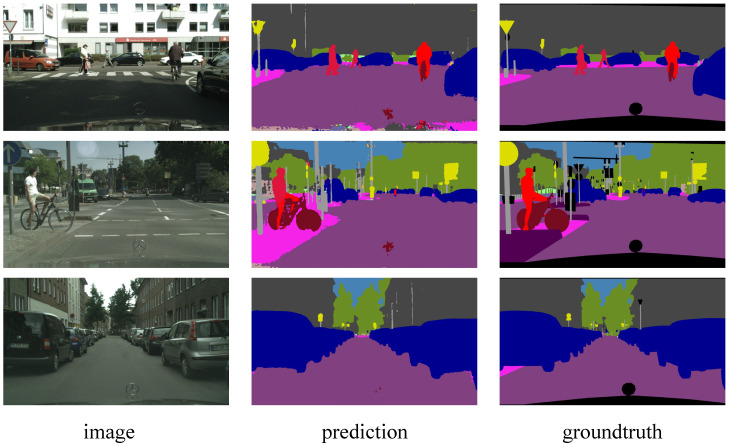
Visualization results of SCGNet-L on Cityscapes validation set. Each sample is presented in an image–prediction–groundtruth triplet.

The segmentation resluts of SCGNet-L on the Cityscapes dataset are visualized in Figure 4. Each sample is presented with an image–prediction–groundtruth triplet in a single row. Note that the ground truth at the bottom is the car window taken by the camera, which has no semantic label and therefore does not participate in the loss calculation. It can be seen that in urban scenes, major semantic categories include vehicles, trees, pedestrians, buildings, and roads. Overall, Spiking CGNet is good at segmenting large-scale regions. The area segmentation is prominent for background semantic categories such as roads, buildings, trees, and sky. For semantic categories of objects, the network can satisfactorily segment the positions of vehicles, pedestrians, and traffic signs, which is beneficial for automatic driving applications on urban roads. The prediction accuracies of different categories are different, which depends mainly on the number of corresponding sample categories in the training set.

### 4.3. Comparisons on DDD17

Spiking CGNet is compared with the other SNNs on the event stream dataset. Before this paper, Spiking FCN and Spiking DeepLab [58] were the only segmenters based on SNN. The comparisons on DDD17 dataset are shown in Table 2. It can be seen that the simulation time step of SCGNet-S and SCGNet-L is 4 are much smaller than Spiking DeepLab and FCN. This indicates that our networks have lower latency and are more suitable to be deployed in mobile devices. Meanwhile, the mIoU of Spiking CGNet is much higher. SCGNet-L achieves 17.22% higher mIoU than Spiking FCN, which is a major breakthrough for SNN in event segmentation.

**Figure 5:**
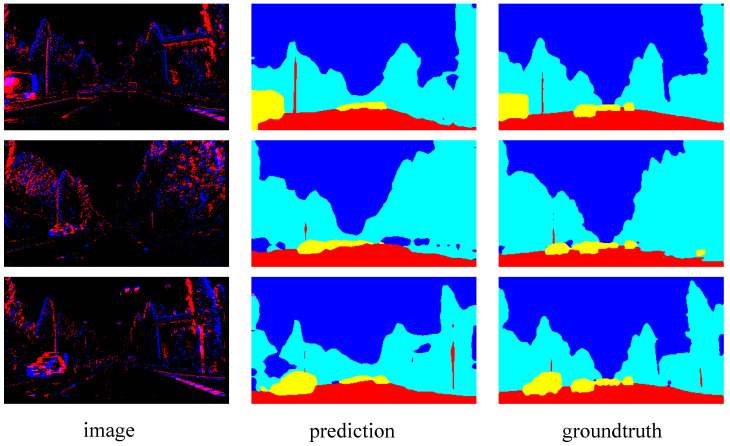
Visualization results of SCGNet-L on the DDD17 test set. The images are visualized version of the events in positive and negative polarities. Each sample is presented in a image–prediction–groundtruth triplet.

The visualization results on the DDD17 dataset are shown in Figure 5. The event images are a visualized version of the events in positive and negative polarities. Positive and negative are presented in red and blue, respectively. Each sample is presented with an image–prediction–groundtruth triplet in a single row. From the ground truth, one can see that the semantic labels of the DDD17 dataset are coarser than that of the Cityscapes dataset. Many close parts are merged into one category, such as road and pavement are merged as flat. The image shows that the outline of objects can be roughly distinguished, such as the background, vehicles, roads, etc. However, the recognition degree of event images is much lower than that of static images in Figure 4, so segmentation on this dataset is obviously more challenging. From the prediction result, as can be observed, SCGNet-L is capable of segmenting the general outline of categories, such as the background and vehicle, quite effectively. However, when focusing on the boundaries between different categories, the reliability of this prediction will be much lower. Overall, Spiking CGNet’s segmentation of event images is a good attempt. To improve the segmentation accuracy, on the one hand, this paper can increase the discrete event resolution and extend the time interval of events. On the other hand, the static and event images can be combined to complement each other in terms of dynamic and stationary objects.

### 4.4. Ablation Studies

#### 4.4.1. Energy Analysis

The main advantage of SNN over ANN is the lower energy consumption, which is also the main advantage of our Spiking CGNet. This advantage mainly comes from two aspects. The first is the difference in energy consumption between MAC and AC operations. Taking standard 45 nm CMOS technology [68] as an example, the energy consumption of different 32-bit float-point operations is shown in Table 3. The energy consumption of AC is only about one-fifth that of MAC, and this gap is much larger on neuromorphic chips. The second is that SNN is a sparse network, and AC operation is only required when a neuron fires a spike, as in Equation (12).

**Figure 6:**
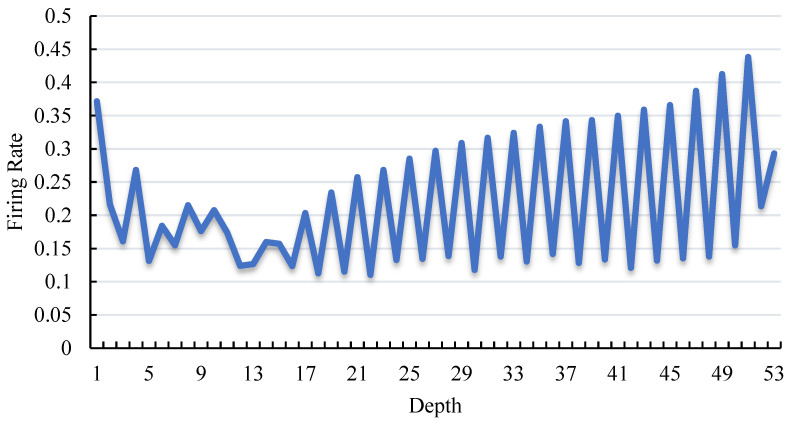
Firing rate of spiking neurons across all layers in SCGNet-S. The depth denotes the layer index of spiking neurons. The firing rate refers to the proportion of all neurons which fire a spike.

The layer-wise firing rate of SCGNet-S on the Cityscape dataset is shown in Figure 6. As can be seen, the firing rate of neurons is generally maintained at a low level. The highest firing rate is less than 45%, and the average firing rate is 21.7%. The first three convolutional layers of the network are sequentially connected without skip connections, so the firing rate decreases when the depth is less than 3. The depth of stage1 grows from, and stage2 contains layers deeper than 11. Globally, the firing rate of stage1 and stage2 is continuously increasing. Due to the membrane shortcut mechanism, the potential residual accumulates as it goes deeper. Locally, the curve is jagged because there is no residual connection in the middle layer of a single SCG block, and the activation leaking caused by spiking neurons is relatively significant.

Using 3×512×1024 static images as input, this paper analyzes the energy consumption of ANN CGNet and our Spiking CGNet. Since batch normalization (BN) can be incorporated into the convolutional layers during inference, the influence of BN layers is ignored. Analysis results are shown in Table 4.

As expected, most of the calculations in SNN are AC operations. This paper uses $E_{ANN}/E_{method}$ in the last column to demonstrate energy efficiency. The energy efficiency of SCGNet-S is 4.92 × that of ANN CGNet. Furthermore, the energy efficiency of SCGNet-L is 1.23 × that of ANN CGNet. With 1.75% higher mIoU, it has been proven that SCGNet-L is more suitable for mobile applications in terms of both accuracy and energy efficiency.

#### 4.4.2. Effect of Global Context Extractor

As mentioned in Section 3.3.2, the global context extractor refines joint features with an attention-like structure. Although non-spike computation (MAC) is used, this module is retained. Here, this paper demonstrates the impact of the GCE module on energy consumption and mIoU, as is shown in Table 5.

It can be seen that the GCE module brings a $1.3\times 10^{8}$ pJ (about 2.01% of the entire network) energy burden to the network, but it brings a 1.44% increase in mIoU, which is satisfying. As a comparison, the energy consumption of SCGNet-L is four times that of SCGNet-S, but the mIoU has only increased by 4.44%.

## 5. Conclusions and Future Works

In this paper, a spiking segmenter is proposed with substantially lower energy consumption and comparable performance for both frame and event-based images. Utilizing the spiking neurons and membrane shortcut, this paper develops a novel spiking context-guided block with spike computations. Furthermore, this paper establishes the spiking context-guided network with well-designed spike encoding and decoding layers. Experiments on the Cityscapes and DDD17 datasets show high energy-efficiency and performance. On the static dataset Cityscapes, the proposed SCGNet-L achieved a mIoU of 66.5%, which is 1.7% higher than ANN CGNet with 1.29 × higher energy-efficiency. On the event dataset DDD17, SCGNet achieve a mIoU of 51.42%, which is much higher than the 34.20% of the previous method spiking FCN.

In summary, spiking neural networks have the potential to achieve better performance with lower energy consumption. This work is a good practice of deep SNNs in semantic segmentation, which may promote the practical applications of SNNs.

For future work, research on semantic segmentation algorithms will be continued based on spiking neural networks. On the one hand, the fusion of information will be investigated from the frame and event-based images and design semantic segmentation networks with multi-modality inputs to further improve the accuracy. On the other hand, future research could start from the structure-level techniques and complete semantic segmentation tasks more efficiently based on more advanced spiking structures such as spiking transformers.

## Figures and Tables

**Table 1:** Comparisons about network type, parameters, FLOPS, and mIoU on the Cityscapes test set. Our Spiking CGNet is compared with other ANN real-time semantic segmentation networks.

Method	Network Type	Time Steps	Parameters (M)	FLOPS_MAC(G)	FLOPS_AC(G)	mIoU (%)
SegNet [63]	ANN	-	29.5	286.0	0	56.1
ENet [60]	ANN	-	0.4	3.8	0	58.3
ERFNet [64]	ANN	-	2.1	21.0	0	68.0
ESPNet [65]	ANN	-	0.4	4.0	0	60.3
BiSeNet [25]	ANN	-	5.8	5.2	0	68.4
BiSeNetV2 [26]	ANN	-	-	16.5	0	72.6
CABiNet [30]	ANN	-	2.6	2.3	0	75.9
HyperSeg-S [66]	ANN	-	10.2	5.9	0	78.1
DDRNet-39 [27]	ANN	-	32.3	54.9	0	80.4
PIDNet-L [28]	ANN	-	36.9	53.9	0	80.6
CGNet [1]	ANN	-	0.5	6.0	0	64.8
SCGNet-S (ours)	SNN	4	0.5	0.1	5.1	62.5
SCGNet-L (ours)	SNN	4	1.9	0.2	20.2	66.5

**Table 2:** Simulation time steps and mean IoU of spiking neural networks on the DDD17 dataset.

Method	Time-Steps	mIoU (%)
Spiking DeepLab	20	33.70
Spiking FCN	20	34.20
SCGNet-S (ours)	4	49.27
SCGNet-L (ours)	4	51.42

**Table 3:** Energy comsumption of 32-bit floating-point operations.

Operation	Energy (pJ)
MULT	3.7
ADD	0.9
MAC (MULT + ADD)	4.6
AC (ADD)	0.9

**Table 4:** Energy comparison between ANN CGNet and Spiking CGNet. The number of MACs and ACs are calculated with a 3×512×1024 static image as input. In addition, the energy consumption is calculated referring to the 45 nm 32-bit float-point operation. $E_{ANN}/E_{method}$ in the last column demonstrates the relative energy efficiency.

32bit-FP: MAC 4.6 pJ AC 0.9 pJ							
**Method**	**Network Type**	**Parameters**	**MACs**	**ACs**	**Energy**	$E_{ANN}/E_{method}$	**mIoU**
CGNet	ANN	0.50 M	6.87 G	0 G	$3.16\times 10^{10}$ pJ	1× (reference)	64.80
SCGNet-S (ours)	SNN	0.49 M	0.14 G	6.50 G	$6.50\times 10^{9}$ pJ	**4**.85×	62.50
SCGNet-L (ours)	SNN	1.85 M	0.28 G	25.89 G	$2.46\times 10^{10}$ pJ	1.29×	66.55

**Table 5:** The effectiveness and energy analysis for global context extractor.

Network	With GCE	Energy	mIoU
SCGNet-S	**✗**	$6.37\times 10^{9}$ pJ	61.06
SCGNet-S	**✓**	$6.50\times 10^{9}$ pJ	62.50

## Data Availability

This paper uses the public dataset Cityscapes and DDD17, which are openly available at https://www.cityscapes-dataset.com/downloads/ (accessed on 1 May 2023) and https://sensors.ini.uzh.ch/news_page/DDD17.html (accessed on 15 May 2023). The semantic label of DDD17 dataset is provided at https://github.com/uzh-rpg/ess.git (accessed on 15 May 2023). No new data were created or analyzed in this study.

## Abbreviations

The following abbreviations are used in this manuscript:

ANN	Artificial neural network
SNN	Spiking neural network
Spiking CGNet	Spiking context-guided network
CGNet	Context-guided network
DVS	Dynamic vision sensors
PReLU	Parametric rectified linear unit
IF	Integrate-and-fire
LIF	Leaky integrate-and-fire
PLIF	Parametric leaky integrate-and-fire
GCE	Global context extractor
mIoU	Mean intersection over union
MAC	Multiply-and-accumulate
AC	Accumulate
